# Comprehensive genomic analysis identifies pathogenic variants in maturity-onset diabetes of the young (MODY) patients in South India

**DOI:** 10.1186/s12881-018-0528-6

**Published:** 2018-02-13

**Authors:** Viswanathan Mohan, Venkatesan Radha, Thong T. Nguyen, Eric W. Stawiski, Kanika Bajaj Pahuja, Leonard D. Goldstein, Jennifer Tom, Ranjit Mohan Anjana, Monica Kong-Beltran, Tushar Bhangale, Suresh Jahnavi, Radhakrishnan Chandni, Vijay Gayathri, Paul George, Na Zhang, Sakthivel Murugan, Sameer Phalke, Subhra Chaudhuri, Ravi Gupta, Jingli Zhang, Sam Santhosh, Jeremy Stinson, Zora Modrusan, V. L. Ramprasad, Somasekar Seshagiri, Andrew S. Peterson

**Affiliations:** 1 Madras Diabetes Research Foundation & Dr. Mohan’s Diabetes Specialities Centre, No. 4, Conran Smith Road, Gopalapuram, Chennai, Tamil Nadu 600 086 India; 2 Department of Molecular Biology, Genentech Inc., 1 DNA Way, South San Francisco, CA 94080 USA; 3 Department of Bioinformatics and Computational Biology, Genentech Inc., 1 DNA Way, South San Francisco, CA 94080 USA; 4 Department of Human Genetics, Genentech Inc., 1 DNA Way, South San Francisco, CA 94080 USA; 5 Department of General Medicine, Govt. Medical College, Kozhikode, 673008 India; 6 MedGenome, Bangalore, Karnataka 560 099 India

**Keywords:** MODY, Diabetes, Exome, Genomics analysis, NKX6–1

## Abstract

**Background:**

Maturity-onset diabetes of the young (MODY) is an early-onset, autosomal dominant form of non-insulin dependent diabetes. Genetic diagnosis of MODY can transform patient management. Earlier data on the genetic predisposition to MODY have come primarily from familial studies in populations of European origin.

**Methods:**

In this study, we carried out a comprehensive genomic analysis of 289 individuals from India that included 152 clinically diagnosed MODY cases to identify variants in known MODY genes. Further, we have analyzed exome data to identify putative MODY relevant variants in genes previously not implicated in MODY. Functional validation of MODY relevant variants was also performed.

**Results:**

We found MODY 3 (*HNF1A*; 7.2%) to be most frequently mutated followed by MODY 12 (*ABCC8*; 3.3%). They together account for ~ 11% of the cases. In addition to known MODY genes, we report the identification of variants in *RFX6*, *WFS1*, *AKT2*, *NKX6–1* that may contribute to development of MODY. Functional assessment of the *NKX6–1* variants showed that they are functionally impaired.

**Conclusions:**

Our findings showed *HNF1A* and *ABCC8* to be the most frequently mutated MODY genes in south India. Further we provide evidence for additional MODY relevant genes, such as *NKX6–1*, and these require further validation.

**Electronic supplementary material:**

The online version of this article (10.1186/s12881-018-0528-6) contains supplementary material, which is available to authorized users.

## Background

Maturity-onset diabetes of the young (MODY) refers to a group of monogenic, non-insulin dependent forms of diabetes that affect non-obese young adults with a prevalence of ~ 100 per million in European populations [1, 2]. MODY has a high inheritance rate and represents one end of a continuum of monogenic forms of diabetes that includes neonatal diabetes [1–3]. Seven of the known MODY genes, the largest functional class, encode transcription factors which are involved in development, maturation and maintenance of β-cells while the remaining are primarily involved in the process of insulin release in response to glucose [1–3]. Although molecular genetic studies have identified heterozygous causal variants in 14 MODY genes, it is believed that additional MODY genes remain to be identified [1, 4].

MODY is often misdiagnosed as either type 1 or type 2 diabetes [5]. Thus, an accurate molecular genetic diagnosis of monogenic diabetes forms like MODY can have a significant impact on patient care. For example, individuals with mutations in *GCK* do not ordinarily need pharmacological treatment whereas those with mutations in *KCNJ11* or *ABCC8* are often best managed with high dose sulfonylureas rather than with insulin, and those with mutations in *HNF1A* or *HNF4A* benefit from treatment with low dose sulfonylureas [6, 7]. A recent study on diabetes in youth reported that MODY was misdiagnosed in 36% of the cases as type 1 (T1DM) and in 51% of the cases as type 2 (T2DM), highlighting the need for the inclusion of molecular genetic diagnosis in the management of young diabetic patients [8].

Given the opportunity for accurate diagnosis and informed treatment, genetic testing for MODY is an ideal test case for effective implementation of precision health care [1, 7]. For those individuals whose age of onset is less than 30 years, molecular genetic testing for variants in known MODY genes has the potential to provide significant patient benefit [6]. For those with later onset or for those who are young and asymptomatic, it is not clear whether genotyping would be beneficial since most studies of MODY have focused on affected individuals and closely related family members, leading to inflated estimates of effect sizes. A recent study of 7 MODY genes in ~ 4000 individuals of European and African-American ancestry identified variants that are predicted to be damaging at a general population frequency of 0.5–1.5% [9]. Damaging variants were however substantially enriched in cohorts of MODY patients or in young, lean subjects with diabetes thus reiterating the potential value of genetic diagnosis in guiding treatment for those diagnosed with diabetes by conventional criteria. A significant caveat to the interpretations outlined above is that the overwhelming majority of sequencing studies that seek to understand disease-causing variants and estimate disease burden have focused on individuals of Northern European descent [10]. These studies have found that ~ 70–80% of causal variants are in either *GCK* or *HNF1A*. Small-scale studies of South Asian MODY patients have indicated that substantially fewer MODY cases can be explained by variants in *HNF1A* [11, 12], *HNF4A* [13], *GCK* [14] and *HNF1B* [15]. Given the distinct clinical profile of diabetes in the Indian subcontinent [16, 17] it is not surprising that a different pattern of MODY inheritance may exist and indeed South Asians may harbor variants in yet to be identified MODY loci. The large population, the significant burden of Mendelian disorders, the very high incidence of diabetes and the earlier age of onset in the Indian subcontinent [18] make it particularly important to understand the pattern of MODY gene variation in India.

In this study, we have performed a comprehensive characterization of the spectrum of MODY gene variation in a cohort of patients from South India. Our genomic analysis and functional testing indicate *NKX6–1* to be a potential MODY gene.

## Methods

### Samples, DNA and RNA preps

Diabetic subjects were recruited from a large diabetes center in Chennai (formerly Madras) city in southern India. All patients underwent a structured assessment including detailed family history and met MODY clinical criteria of Fajans and Tattersal [19]: age at diagnosis of diabetes 30 years or less, control of hyperglycemia for a minimum period of 2 years without insulin, negative for auto antibodies, absence of ketonuria at any time and evidence of autosomal dominant inheritance including a three-generation family history of diabetes.

Clinical characteristics of the MODY patients and those of the normal glucose tolerant controls are given in Additional file 1: Table S1a. Written informed consent was obtained from all the study participants. The study was approved by the Institutional Ethics Committee of the Madras Diabetes Research Foundation. The reported investigations have been carried out in accordance with the principles of the Declaration of Helsinki.

Consented and de-identified patient blood samples were used for extraction of DNA. EDTA anti-coagulated venous blood samples were collected from all study subjects, and the genomic DNA was isolated from whole blood by proteinase K digestion followed by phenol-chloroform extraction. Subsequently genomic DNA was precipitated in ethanol. The quality and quantity were assessed spectrophotometrically.

A total of 289 samples were used in this study. This consisted of 152 clinically diagnosed MODY cases and 137 normal glucose tolerant (NGT) subjects used for variant filtering.

### Whole genome, exome and targeted sequencing

Whole genome libraries were constructed using TruSeqNano DNA Library Preparation Kit (Illumina, CA) and sequenced on Illumina HiSeq2500 (Illumina, CA) to generate 2 × 75 bp read.

The whole exome analysis was performed using Agilent SureSelect (Santa Clara, CA) Human All Exome kit v5 (50 Mb). Exome capture libraries were sequenced on HiSeq 2500 (Illumina, CA). Targeted exome sequencing of 258 samples (includes 121 MODY samples and 137 normal glucose tolerant subjects) was performed using custom probes corresponding to 1965 genes that included established MODY genes and those that were implicated in pancreatic cell biology and/or diabetes (Additional file 1: Table S3).

### Sequence data processing

All sequencing reads were evaluated for quality using the Bioconductor ShortRead package [20]. An all-against-all sample comparison was done on germline variants to confirm the patient exome-WGS data pairing and establish sample relatedness prior to additional data analysis.

### Variant calling

Whole genome and whole exome sequencing data were combined and then processed using the Genome Analysis Toolkit (GATK) (version v3.5–0-g36282e4) best practices recommendations [21, 22]. Briefly, reads were mapped to the human reference genome GRCh37 using BWA-MEM (version 0.7.10; http://arxiv.org/abs/1303.3997). Duplicate alignments were marked and removed using Picard tool (version 1.126) (http://broadinstitute.github.io/picard/) followed by indel realignment and base quality score recalibration. The GATK Haplotype Caller algorithm was used to generate gVCFs for all samples. Joint variant calling was performed separately for discovery cohort samples and validation cohort samples using GATK Genotype GVCFs. Variant quality score recalibration was carried out to estimate the confidence of variants called in the discovery cohort. For the validation cohort, standard hard filters were applied using a set of criteria and parameters as recommended by GATK [22]. Variant annotation was carried out using SnpEff (version 4.1) [23].

### Variant filtering

Our variant filtering strategy is outlined in Fig. 2a. It involved sequential filtering steps followed by manual review. We filtered for variants that meet the following three criteria: variants that (1) were rare (MAF < = 1% in 1000 genomes [24] and the NHLBI Exome Sequencing Project (ESP) [25]; (2) were protein-altering or potentially protein-altering; (3) and were in the curated list of 35 genes that included known MODY genes and others implicated in early onset diabetes including neonatal diabetes (Additional file 1: Table S3). In the manual review process, the set of variants obtained following filtering were manually checked for read evidence using Integrative Genomics Viewer (IGV) [26]. We retained ultra-rare variants, which have an allele-frequency < 0.01% in ExAC and were present in at the most only one sample in the ExAC South Asian cohort. Finally, we checked if any of our filtered variants were reported in previous MODY studies as disease relevant and annotated them.

### Plasmids, stable cell lines and expression studies

Mutant *NKX6-1* constructs were generated using a site-directed mutagenesis kit (Stratagene, USA) in TOPO-TA vector backbone. The mutant and wildtype sequences were sub-cloned from TOPO-TA vector into pLVX-TetOne-Puro vector (Clonetech, USA; Cat No. 631847) or pRK5 vector (Genentech, CA). Lenti-X 293T packaging cells (Clontech, USA; Cat No. 632180) were transfected with clones carrying variants in the pLVX-TetOne-Puro vector using Lenti-X Packaging Single Shots (VSV-G) (Clontech, USA; Cat No. 631275). At 48h post transfection, lentivirus containing supernatants were used to infect β-TC-6 cells according to the standard protocol in the Lenti-X Tet-One Inducible Expression Systems User Manual (Clontech, USA) for 24 h. 2 μg/mL puromycin (Gibco, USA; Cat No. A11138–03) containing β-TC-6 complete growth media with Tet-free FBS (Clontech, USA; Cat No. 631101) was used to select infected cells for 7 days. Post selection, β-TC-6 stable cell lines were maintained in complete growth media supplemented with 0.5 μg/mL puromycin. All the stable cell lines used in the subsequent studies were passaged for at least 3 times and tested with Lenti-X p24 Rapid Titer Kit (Clontech, Cat No. 632200) for the clearance of lentivirus. Stable cell lines were expanded and cultured in complete growth media with 15% Tet-free FBS for 24 h. We seeded 50,000 cells in a 12 well plate and induced gene expression using 100 ng/ml doxycycline (dox). Cells were harvested immediately following induction (0 h) and at 48 h post induction. Cells were lysed and total RNA from cell lines was extracted using RNeasy Plus Mini kit (Qiagen, CA). For each stable cell line expressing a particular gene we prepared three biological replicates for each condition (wt and variants) at 0 h (no Dox) and 48 h (with Dox) time point.

### RNA-Seq and data analysis

We used 0.5μg of total RNA to generate RNA-seq libraries using TruSeq RNA Sample Preparation kit (Illumina, CA). The libraries were multiplexed and sequenced on HiSeq 2500 to obtain on average ~ 30 million single end 50 bp reads per sample.

RNA-seq data from mouse cell lines with human expression constructs were mapped to the mouse genome (GRCm38/mm10) and respective wildtype human construct. Mapping was performed with GSNAP (2013–10-10) [27]. Only uniquely mapped reads were considered for downstream analysis. Gene models were based on RefSeq transcripts and NCBI gene annotation. Differential expression was performed with the R/Bioconductor package DESeq2 [28]. For clustering, count data were transformed by variance stabilization and genes were centered to have mean zero. Clustering was performed using 1-Pearson correlation as distance metric and average linkage. Heat maps were generated using the R/Bioconductor package NMF.

### Western blot analysis

Expression of NKX6–1 was tested using western blot as previously described [29]. Briefly, β-TC-6 stable cells (50,000) expressing NKX6–1 were harvested at 48 h post induction with 100 ng/ml dox. Lysates were prepared for western blotting as previously described [29]. We used anti-NKX6–1 (Novus Biologicals; NBP-149672) and anti-Hsp90 (Santa Cruz Biotechnology; sc-7947) antibodies for protein detection. Appropriate HRP-conjugated secondary antibodies (Pierce Biotechnology, IL) were used along with the primary antibodies in the western blot assay.

## Results

### Clinical characteristics

**Fig. 1:**
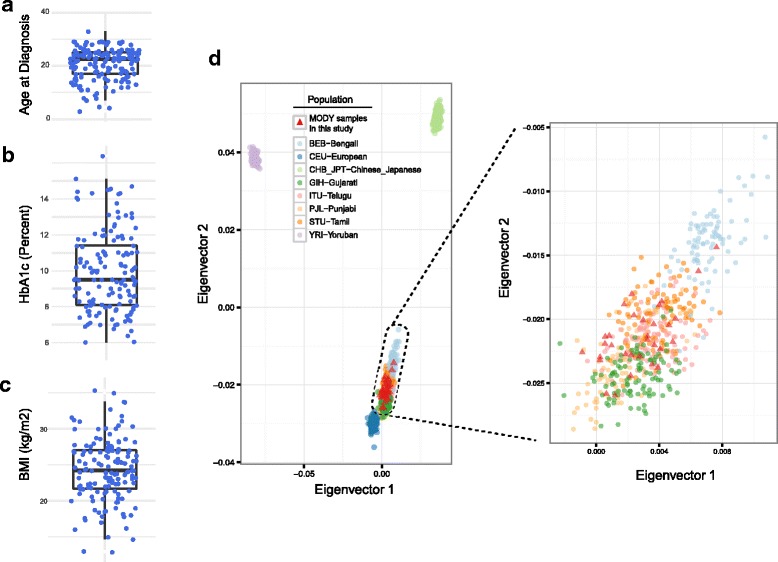
MODY samples. **a-c** Box plots of age at ascertainment or diagnosis of diabetes (**a**), HbA1c (**b**) and BMI (**c**) for MODY patients; (**d**) PCA analysis of 31 MODY patient exome samples shown along with samples from 1000 Genomes Phase 3 data. The median value is shown as a line with the whiskers extending from the highest value within 1.5 * IQR of the third quartile to the lowest value within 1.5 * IQR of the first quartile where IQR is the inter-quartile range

Our genomic analysis included 152 clinically diagnosed MODY patients (Additional file 1: Table S1a). The mean age of diabetes ascertainment for patients in our cohort was 20.85 ± 5.9 (±SD) years (Fig. 1a). This included 56 female and 96 male patients. Mean HbA1c glycated haemoglobin (HbA1c) values were 8.95 ± 2.26% (Fig. 1b) and mean BMI of these patients was 24.29 ± 4.37 kg/m^2^ (Fig. 1c). Additional clinical characteristics of the discovery cohort (Additional file 1: Table S1b and Additional file 2: Figure S1) include fasting plasma glucose (mean: 166.2 ± 66.85 mg/dL) and fasting C-peptide levels (mean: 0.668 ± 0.40 ng/mL) and post breakfast stimulated C-peptide level (1.60 ± 0.73 ng/mL).

### Identification of MODY relevant variants

We performed whole or targeted exome sequencing of our 152 MODY cases to assess the presence of MODY relevant variation. All samples were confirmed to be unrelated (Additional file 2: Figure S2). We initially performed whole exome sequencing on 31 MODY probands (Additional file 1: Table S1a). We also performed low-pass whole genome sequencing (mean coverage ~ 6×) of the 31 probands. We combined reads from exome and low-pass WGS and achieved > 46× coverage for the 31 samples (Additional file 1: Table S2a). Principal component analysis [30] using the genotypes called from low-pass WGS, merged with those from 1000 Genomes [24] project phase 3 samples, showed that the 31 samples overlap with Indian reference samples, as expected (Fig. 1d) confirming the ethnicity of the samples used in the study. We assessed the remaining 121 MODY patient samples for presence of pathogenic variants using targeted exome sequencing (see Methods). We obtained an average coverage of 82× for the 121 samples (Additional file 1: Table S2b).

**Fig. 2:**
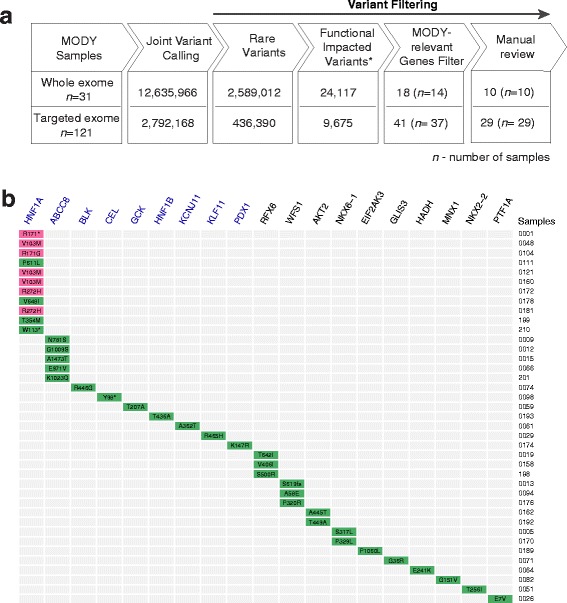
MODY relevant variant identification. **a** Schematic representation of the steps involved in MODY relevant variant identification; * indicates variants that are protein-altering or potentially protein-altering. **b** MODY relevant variants identified in indicated genes for each patient sample. Pink indicates variants previously reported, green indicates novel or ultra-rare variants

To identify MODY relevant variants we started with 12,635,966 variants that resulted from the joint variant calling across 31 probands (see Methods; Fig. 2a**)**. We filtered out common variants present at MAF > = 1% frequency in 1000 genomes [24] or the NHLBI Exome Sequencing Project (ESP) [25] to obtain 2,589,012 variants. We next focused on 24,117 variants predicted to have an impact on protein function and/or disease relevance. We discarded variants present in the 137 NGT samples. To facilitate variant interpretation, we focused on analysis of variants in a curated list of 35 genes that included known MODY genes and others implicated in early onset diabetes including neonatal diabetes. This resulted in 18 candidate MODY variants in 12 genes in 14 of the 31 probands (Fig. 2a). Finally, we performed manual review (see Method) on this variant list and arrived at 10 candidate variants (Fig. 2a**;** Additional file 1: Table S4).

Targeted exome sequencing and analysis of the 121 MODY samples resulted 2,792,168 variants. We applied the same filtering strategy as done for the whole exome samples above and finally arrived at 29 variants (Fig. 2a**;** Additional file 1: Table S4).

Combined together, we identified 39 candidate variants in 39 of the 152 MODY samples (Fig. 2b**;** Additional file 1: Table S4). Of these, variants R272H and V103M found in HNF1A were observed in two and three patient samples, respectively (Fig. 2b). Of the 14 known MODY genes [1], 9 were found mutated in our samples (Fig. 2b). *HNF1A* (MODY 3) was most frequently mutated at 7.2% (11/152), followed by *ABCC8* at 3.3% (MODY 12; 5/152). Other MODY genes were found to be mutated in one sample and this includes *BLK* (MODY 11), *CEL* (MODY 8), *GCK* (MODY 2), *HNF1B* (MODY 5), *KCNJ11* (MODY 13), *KLF11* (MODY 7), and *PDX1* (MODY 4). In addition to the known MODY genes, we found candidate variants in 10 MODY-relevant genes. This includes *RFX6* (*n* = 3), *WFS1* (n = 3), *NKX6–1* (*n* = 2), *AKT2* (n = 2) (Fig. 2b**;** Additional file 1: Table S4). All variants identified, except for *WFS1* P320R, were heterozygous, consistent with the dominant inheritance pattern of MODY.

**Fig. 3:**
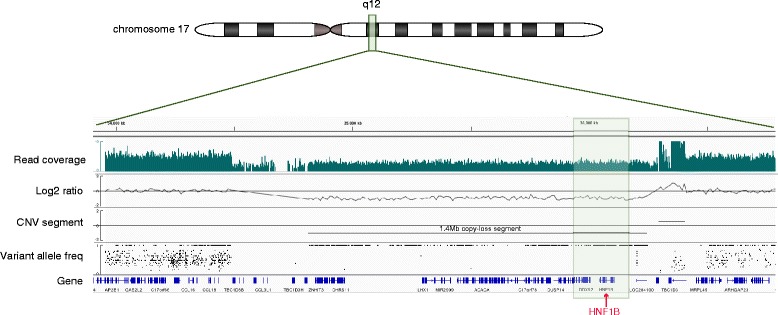
HNF1B deletion in a MODY patient. Copy number analysis of MODY sample MDX 0042

Besides single-nucleotide variants, we assessed the WGS data for copy loss in known MODY genes in the 32 patients with WGS data. We detected copy loss in proband MDX 0042 that spans a 1.4 Mb region of 17q12 that contains *HNF1B* and is flanked by *TBC1D3G* at the 5′ end *TBC1D3F* at the 3′ end (Fig. 3). This patient had no other MODY candidate variants, suggesting that the loss of one copy of HNF1B is responsible for the phenotype.

### NKX6–1 variants found in MODY patients are functionally impaired

**Fig. 4:**
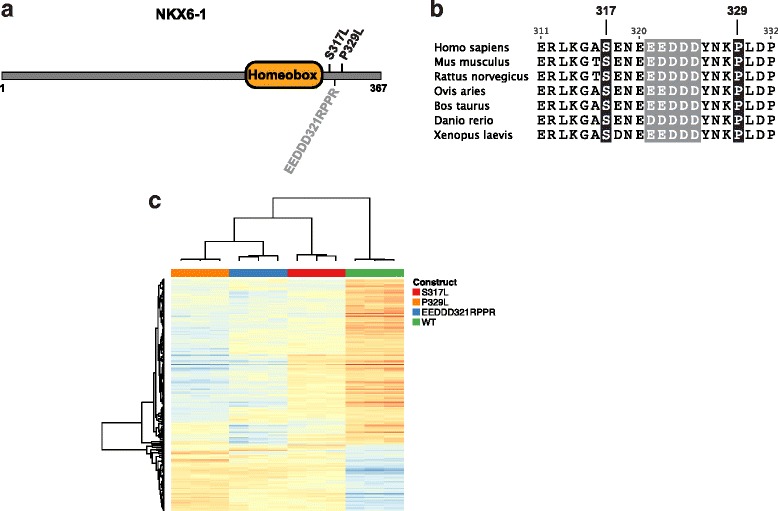
Functional analysis of NKX6–1 variants. **a** MODY relevant NKX6–1 variants. Numbers indicate amino acid position. **b** Multiple sequence alignment of NKX6–1 from the indicated species. **c** Heatmap of differentially expressed genes identified by comparing each mutant against wild-type in cells expressing the indicated variant or wildtype NKX6–1. EEDDD321RPPR is a control variant [33]

Although the variants in *NKX2–2* [31], *RFX6* [32] and *MNX1* [31] have been found in recessively inherited neonatal diabetes, variants in *NKX6–1* have not thus far been found in neonatal diabetes or MODY. To understand these *NKX6–1* variants, we functionally assessed the transcriptional activity of the *NKX6–1* variants by stably expressing them or the wild-type gene in a mouse insulinoma cell line (β-TC-6), using a tetracycline inducible expression system (Additional file 2: Figure S3). We used RNA-seq to assess transcriptional activity of the two NKX6–1 variants, S317L and P329L (Fig. 4c). As a control, we included a previously described and functionally impaired control variant (EEDD321RPPR) [33] (Fig. 4). We confirmed the expression of the NKX6–1 variants (Additional file 2: Figure S3 and Additional file 2: Figure S4).

For each of the two NKX6–1 variants, and the functionally impaired control mutant, we found significant differences in gene expression between cells expressing the variant as compared to cells expressing wild type NKX6–1 (adjusted *p* < 0.05, fold-change > 1.5; Additional file 1: Table S5). Notably, clustering of samples based on genes identified as differentially expressed in at least one of the three comparisons showed that the transcriptional activity of the two MODY relevant variants was similar to the functionally impaired control mutant (Fig. 4c). This suggests that S317L and P329L NKX6–1 variants were functionally impaired and that disruption of the carboxy-terminal domain of NKX6–1 is likely responsible for MODY in these individuals (Fig. 4c). While the function of the carboxy terminus of NKX6–1 is poorly understood, deletions and substitutions have been shown to disrupt the ability of the nearby homeobox domain to bind DNA with appropriate specificity [34].

We found 39 genes that were differentially regulated in common between the two NKX6–1 MODY mutants (Additional file 1: Table S5), suggesting that some of these may be related to the development of diabetes in these patients. Further, inspection of the genes that are dysregulated by the two variants and comparison to NKX6–1 binding sites in the β-TC-6 cell line (MKB, JZ and ASP, unpublished) revealed a number of genes which may be related to the development of diabetes in these patients **(**Additional file 1: Table S5). For example, *BHLAHA15* (MIST1), with a NKX6–1 binding site upstream of the transcription start site, is significantly down-regulated by both variants. *BHLHA15* knockout mice have decreased expression of Glut2 in pancreatic β-cells and age-related impairment in glucose clearance [35], suggesting that its down-regulation could contribute to the development of diabetes in these patients. Interestingly, the most significantly up-regulated gene for all three variants was *MAP3K15* (ASK3). The function of *MAP3K15* has not been studied in any detail but it is highly homologous to Apoptosis-Signal-regulated Kinase 1 (*ASK1*), a gene that stimulates apoptosis in response to ER stress and other noxious stimuli [36], suggesting that an increased rate of apoptotic β cell loss may also be involved in the development of diabetes in patients carrying the *NKX6–1* variants.

## Discussion

Overall, we analyzed 152 clinically diagnosed unrelated MODY patients from southern India for the presence of variants in known MODY genes and additional loci of potential MODY-relevance. We found relevant variants in previously described MODY genes in ~ 15% (23/152) of the cases analyzed (Fig. 2b). As has been previously reported, the locus diversity of MODY in India is unlike that in European populations where up to 80% of patients carry variants in *HNF1A* and *GCK*. We found *HNF1A* variants in ~ 7% of the patients and *GCK* alterations in < 1% of the patient samples, a substantially lower rate than in Europeans and consistent with previous reports of Indian MODY patients [11, 12, 14, 37–39]. We found ~ 3% of the samples carried an *ABCC8* variant (MODY 12). The *ABCC8* variants have important treatment implications, as it has been suggested that these patients may be best managed with the use of sulfonylureas [40, 41], similar to the situation with cases of neonatal diabetes caused by mutations in *KCNJ11* or *ABCC8*.

Importantly, we found 6 variants, including three PDX1 variants P33T [42], E224K [12, 43], P242L [44], HNF4A V169I [12, 45], BLK A71T [12, 46] and NEUROD1 H241Q [12, 47], previously reported to be linked to MODY, to occur at a similar frequencies in the MODY cases and the general population. This suggests that these variants most likely have little to no effect on risk for developing MODY. The findings underscore the difficulty in interpreting relevance of variants where population specific common variant data are not available and highlights the need for such data to enable the application of genomics in the clinic.

In addition to known MODY genes we found rare variants of functional consequence in genes implicated in other forms of diabetes or beta-cell biology. This includes variants in NKX6-1 (S317L, P329L). A role for NKX6–1 in MODY is consistent with its role in β-cell function, maintenance and/or development and the phenotypes described in knockout mice studies [48, 49]. The NKX6-1 S317 and P329 are highly conserved residues across orthologs from multiple organisms and substitution at these positions is likely to impair its normal function. Consistent with this, both S317L and P329 were found to be functionally impaired in their transcriptional activity (Fig. 4). These findings while suggest a plausible role NKX6–1 in MODY further studies would be needed to confirm its role as a MODY gene.

In addition to *NKX6–1* we found variants in *WFS1*, *RFX6* and 7 other genes associated with monogenic forms of diabetes (AKT2, *EIF2AK3, GLIS3, HADH, MNX1, NKX2–2,* and *PTF1A*) and they may be relevant to development of MODY. Additional genetic or functional studies will be required to evaluate and establish the relevance of these genes in MODY.

## Conclusion

In conclusion, our study reports that *HNF1A* and *ABCC8* are among the most frequently mutated MODY genes in south India. Further we provide evidence for additional MODY relevant genes, such as *NKX6–1*, and these require further validation. Our findings have important implications for MODY gene testing.

## Additional files

Additional file 1: Table S1a. Sample information and clinical characteristics. **Table S1b.** Summary of clinical characteristics of MODY patient samples. **Table S2a.** Sequencing statistics of combined exome and WGS. **Table S2b.** Sequencing statistics of targeted exome samples. **Table S3.** Targeted gene panel. **Table S4.** Candidate variants identified in MODY samples. **Table S5.** Differentially expressed genes - NKX6–1 (XLS 292 kb)

Additional file 2: Figure S1. Box plot showing (a) fasting plasma glucose, (b) fasting insulin, (c) C-peptide fasting, (d) C-peptide stimulated and (e) creatinine in MODY and control samples. The median value is shown as a line with the whiskers extending from the highest value within 1.5 * IQR of the third quartile to the lowest value within 1.5 * IQR of the first quartile where IQR is the inter-quartile range. **Figure S2.** Heatmap depicting the genotype based identity of the discovery and validation MODY cohort and control samples. Genomic regions for which we obtained data for the validation cohort samples and corresponding regions from the discovery set samples using GATK joint-variant caller. The sample identity was computed based on the high-confidence set of single nucleotide variants (SNVs) that passed GATK Hard-Filtering criteria. **Figure S3.** Expression level of mouse *Nkx6–1* (top) or human *NKX6–1* (bottom) following induction in cells stably expressing the indicated variant or wildtype. **Figure S4.** Western blot showing the expression of NKX6–1 48 h post dox induction. Hsp90 was used as a loading control. (ZIP 5136 kb)
