# Supplementary material for: Comprehensive genomic analysis identifies pathogenic variants in maturity-onset diabetes of the young (MODY) patients in South India
Source: BMC Med Genet. 2018 Feb 13;19:22. doi: 10.1186/s12881-018-0528-6 (PMC5811965; doi:10.1186/s12881-018-0528-6)
Supplement: Supplementary file 2 — Box plot showing (a) fasting plasma glucose, (b) fasting insulin, (c) C-peptide fasting, (d) C-peptide stimulated and (e) creatinine in MODY and control samples. The median value is shown as a line with the whiskers extending from the highest value within 1.5 * IQR of the third quartile to the lowest value within 1.5 * IQR of the first quartile where IQR is the inter-quartile range. Figure S2. Heatmap depicting the genotype based identity of the discovery and validation MODY cohort and control samples. Genomic regions for which we obtained data for the validation cohort samples and corresponding regions from the discovery set samples using GATK joint-variant caller. The sample identity was computed based on the high-confidence set of single nucleotide variants (SNVs) that passed GATK Hard-Filtering criteria. Figure S3. Expression level of mouse Nkx6–1 (top) or human NKX6–1 (bottom) following induction in cells stably expressing the indicated variant or wildtype. Figure S4. Western blot showing the expression of NKX6–1 48 h post dox induction. Hsp90 was used as a loading control. (ZIP 5136 kb) [file 12881_2018_528_MOESM2_ESM.zip › 03b.SupplementaryFiglegend-11192017R4.docx]

**Supplementary Figure 1.** Box plot showing (a) fasting plasma glucose, (b) fasting insulin, (c) c-peptide fasting, (d) c-peptide stimulated and (e) creatinine in MODY and control samples. The median value is shown as a line with the whiskers extending from the highest value within 1.5 * IQR of the third quartile to the lowest value within 1.5 * IQR of the first quartile where IQR is the inter-quartile range.

**Supplementary Figure 2**. Heatmap depicting the genotype based identity of the discovery and validation MODY cohort and control samples. Genomic regions for which we obtained data for the validation cohort samples and corresponding regions from the discovery set samples using GATK joint-variant caller. The sample identity was computed based on the high-confidence set of single nucleotide variants (SNVs) that passed GATK Hard-Filtering criteria.

**Supplementary Figure 3.** Expression level of mouse *Nkx6-1* (top) or human *NKX6-1* (bottom) following induction in cells stably expressing the indicated variant or wildtype

**Supplementary Figure 4.** Western blot showing the expression of NKX6-1 48h post dox induction. Hsp90 was used as a loading control.
